# Gli1 promotes colorectal cancer metastasis in a Foxm1-dependent manner by activating EMT and PI3K-AKT signaling

**DOI:** 10.18632/oncotarget.13348

**Published:** 2016-11-15

**Authors:** Chuan Zhang, Yong Wang, YiFei Feng, Yue Zhang, Bing Ji, Sen Wang, Ye Sun, Chunyan Zhu, Dongsheng Zhang, Yueming Sun

**Affiliations:** ^1^ Colorectal Department of the First Affiliated Hospital of Nanjing Medical University, Nanjing, Jiangsu CHINA, 210029

**Keywords:** colorectal cancer, Gli1, metastasis, epithelial-to-mesenchymal transition, PI3K-AKT signaling

## Abstract

Colorectal cancer(CRC) is one of the most commonly diagnosed cancers in human beings and metastasis is the main death reason. Recently, Gli1 has been reported to be a key regulator of various cancer biologies and genes expressions. However, the detailed molecular mechanism of Gli1 in CRC metastasis remains largely unknown. In this study, we aimed to investigate the role of Gli1 in CRC metastasis. We used qRT-PCR, Immunohistochemistry and Western blot to test the expression levels of Gli1, Foxm1 and other target genes in the tissues and cells; Lentivirus stable transfection to change the expression levels of Gli1 and Foxm1; Wound-healing, cell invasion, migration assays and tail vein metastatic assay to test the role of Gli1 in CRC metastasis *in vitro* and *vivo*. We demonstrated that Gli1 was significantly overexpressed in colorectal cancer tissues and cells. Foxm1 level had a positive correlation with Gli1. Furthermore, we found that Gli1 promotes colorectal cancer cells metastasis in a Foxm1-dependent manner by activating EMT and PI3K-AKT signaling. Thus, we proved that Gli1 plays important role in CRC metastasis and provided a new visual field on the therapy of CRC metastasis.

## INTRODUCTION

Colorectal cancer is the third most commonly diagnosed cancer in males and the second in females [1]. A large amount of these patients are initially diagnosed with developed stage IV CRC [2], and the underlying mechanism has not been understood well [3]. It is now generally accepted that metastasis is the main cause of death in patients with CRC due to the tumor resistance to conventional therapies and a poor overall survival [4, 5]. Thus, to find the biomarkers of CRC metastasis for its targeted therapy and prognosis is urgently required for clinical medicine.

Hedgehog (Hh) signaling pathway plays a critical role in normal cellular development including embryogenesis, tissue patterning, and differentiation [6]. The first link of Hh signaling to cancer was found in Basal Cell Nevus Syndrome and aberrant Hh signaling activity was also observed in specific types of cancer [7]. In the canonical pathway progression, Hh ligand binds to Ptch1 and releases its tonic inhibition of Smo. Smo then facilitates the activation of Gli transcriptional activators and their translocation into the nucleus to activate expression of Hh target genes [8]. Gli1 is one important transcription factor in classical Hh signaling pathway and activates most of the Hh pathway target genes [9]. But, its role is controversial in CRC, especially in CRC metastasis. Foxm1, a transcriptional factor of the forkhead box family, participates in a wide range of biological processes [10]. More and more studies found that Foxm1 plays an important role in the occurrence and development of malignant tumors and regulate the tumor progression as a downstream target of Gli1 in cancers like basal cell carcinomas and lung carcinomas [11]. But, the underlying mechanism between Gli1 and Foxm1 in regulating the CRC metastasis had not been understood well. In this study, we aimed to investigate the mechanism of Gli1-Foxm1 axis in CRC metastasis. We also explored the crosstalks of Gli1-Foxm1 axis with EMT and EGFR-PI3K/AKT signaling to provide a new visual field on the therapy of CRC metastasis.

## RESULTS

### Gli1 expression had close correlations with the metastasis factors

**Figure 1:**
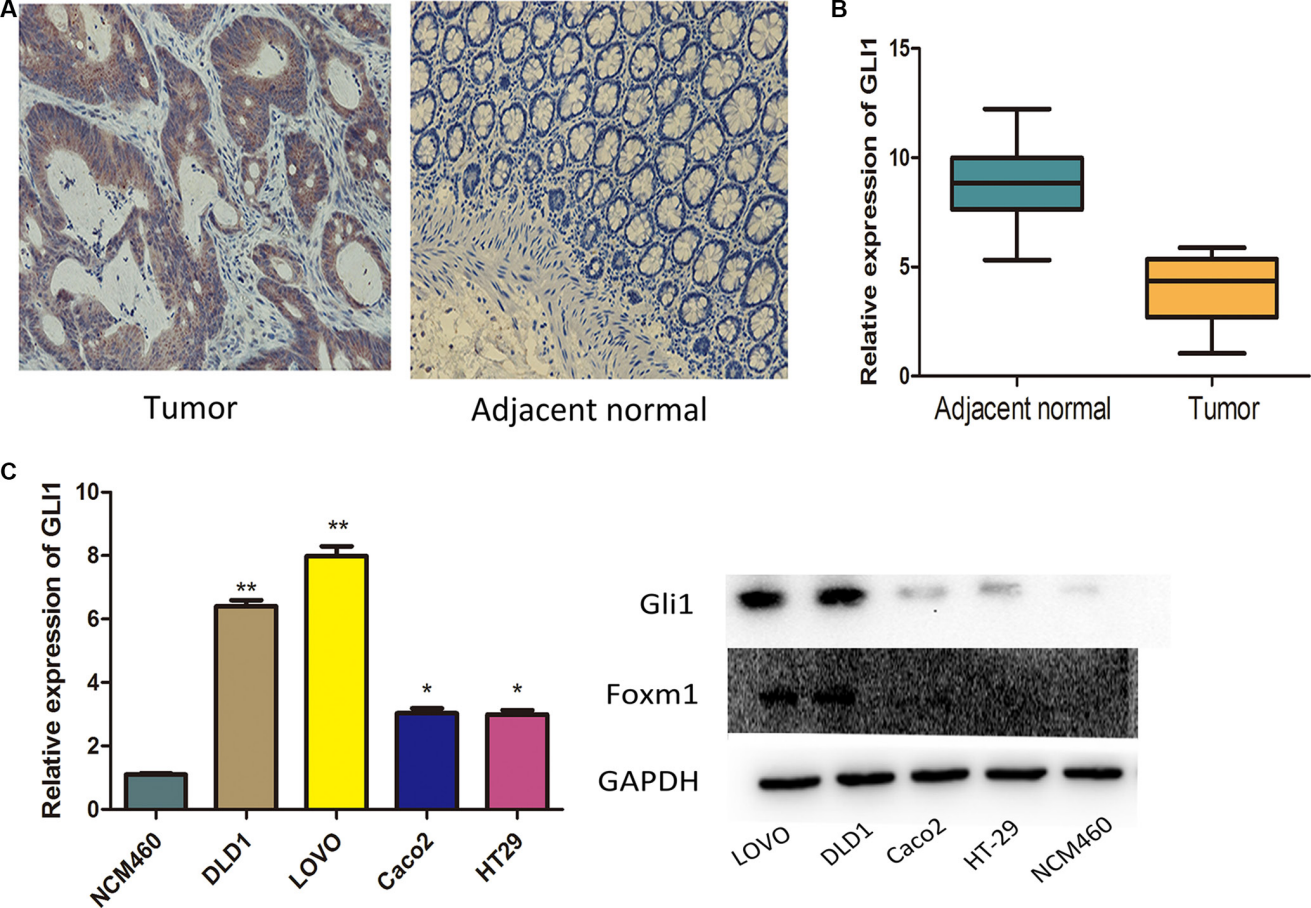
Expression of GLI1 in colorectal cancer tissues and cell lines (**A**) Immunohistochemical results of Gli1 expression in CRC tissues: Gli1 was positively stained in the cytoplasm of the majority of tumor specimens and was negatively or weakly stained in the adjacent normal tissues. (**B**) Gli1 expression levels were assessed in tumor tissues and adjacent normal tissues. The ΔCT value was determined by subtracting the GAPDH CT value from the Gli1 CT value. Smaller ΔCT value indicates higher expression. (**C**) Gli1 expression levels were measured by qRT-PCR and WB together with Foxm1 in CRC cells. The intensity of the bands was determined using densitometric analysis.**p < 0.01, *p < 0.05.

To explore the role of Gli1 in CRC progression, the mRNA expressions were analyzed by qRT-PCR. The level of Gli1 was significantly higher in cancer tissues than adjacent normal tissues, similar with the IHC results (Figure 1A, 1B, Table 1). We divided the patients into two groups by the average Gli1 level: low level and high level, and analysed the tumor Gli1 levels with clinical parameters (Table 2). We found that Gli1 expression had a close correlation with the metastasis factors:higher in patients with nodal metastasis than in those with no metastasis (86.2% vs 67.6%, *P* = 0.02); higher in patients with advanced stage disease than in those with early-stage (84.7% vs 68.7%, *P* = 0.038). Additionally, Gli1 expression had a positive correlation with the CEA level (85.7% vs 61.2%, *P* = 0.002). There were no statistical differences between Gli1 expression and age, gender and T stage.

**Table 1:** Expression of Gli1 in colorectal carcinoma and adjacent normal tissues

Group A			
Specimen	Gli1 IRSs (*N*)	*χ*^2^	*P* value
Carcinoma	4–12 (104)	141.5	< 0.0001
	0–3 (22)		
Adjacent normal	4–12 (10)		
	0–3 (116)		

IRSs, immunoreactive scores. Gli1 was significantly overexpressed in the carcinoma tissues than adjacent normal tissues.

**Table 2:** Association of Gli1 expression and clinical parameters in tumor tissues of colorectal cancer patients

Characteristics	Gli1 levels				*P* value
	Total(*n* = 126)	Low, *n*(%)(*n* = 30)	High,*n*(%)(*n* = 96)		
Age				0.15	0.831
≤ 65	50	11	39		
> 65	76	19	57		
Gender				0.626	0.523
Male	51	14	37		
Female	75	16	59		
T factor				0.01	1
1 + 2	26	6	20		
3 + 4	100	24	76		
N factor				5.944	0.02
0	68	22 (32.4%)	46 (67.6%)		
1 + 2	58	8 (13.8%)	50 (86.2%)		
M factor				5.727	0.012
0	110	30	80		
1	16	0	16		
stage					
I + II	67	21 (31.3%)	46 (68.7%)	4.477	0.038
III + IV	59	9 (15.3%)	50 (84.7%)		
CEA (ng/ml)					
<4.7	49	19 (38.8%)	30 (61.2%)	9.9	0.002
≥4.7	77	11 (14.3%)	66 (85.7%)		

Gli1 in LOVO, DLD1, HT29, Caco2 and normal colon epithelial NCM460 cell lines was then analyzed by qRT-PCR and western blot (WB). The level of Gli1 RNA and protein was significantly higher in LOVO and DLD1 than in HT29, Caco2 and NCM460. But HT29 and Caco2 had no significant differences (Figure 1C, 1D). Thus, we select LOVO to do our study.

### Foxm1 was regulated by Gli1 without a feedback in colorectal cancer

**Figure 2:**
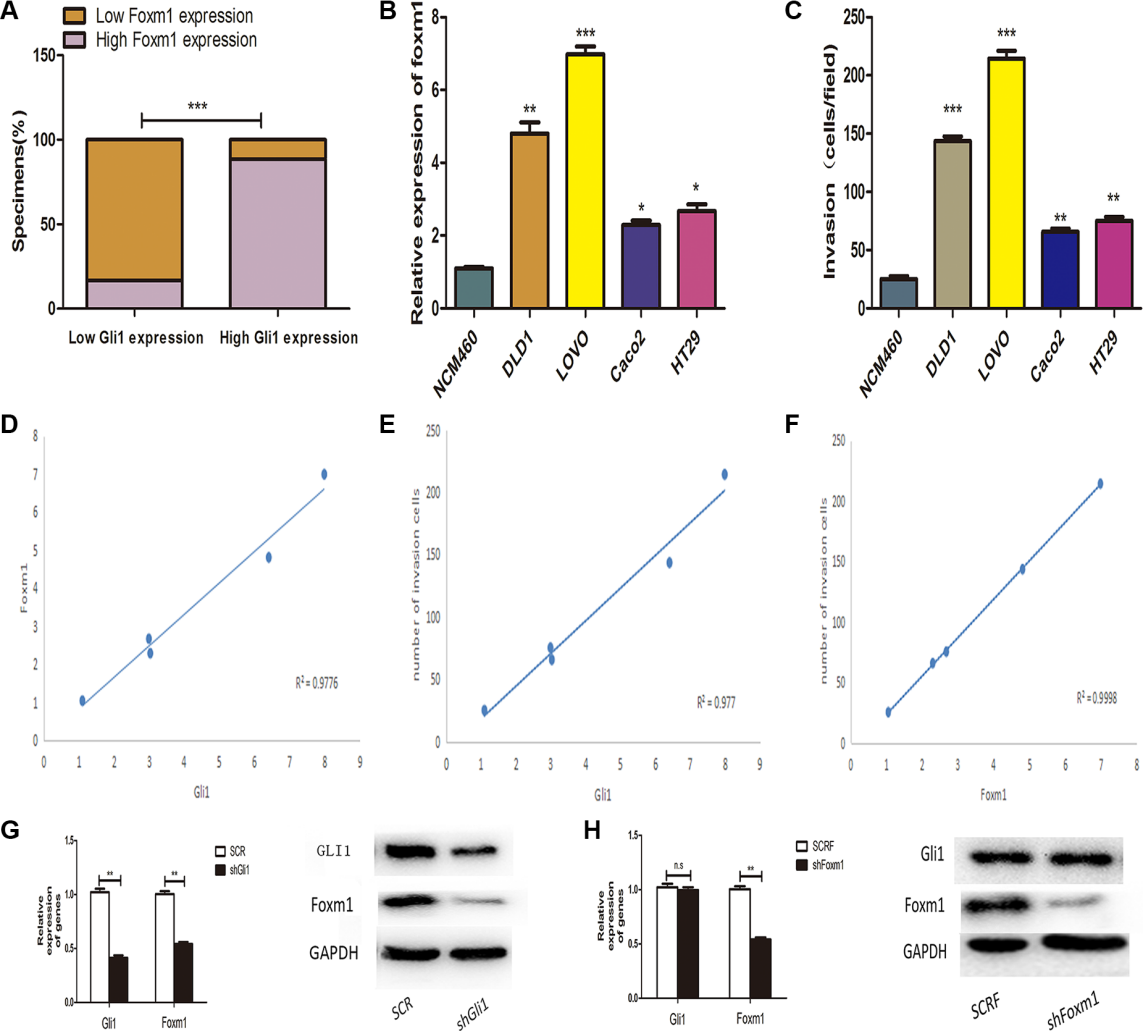
Percentage of specimens exhibiting low or high Gli1 expression and association of Gli1 with Foxm1 in CRC tumor specimens. ***P < 0.001. (**B**) Gli1 expression levels were measured by qRT-PCR. (**C**) The invasive capacity was determined by invasion assays. (**D**) Correlation between the mRNA expression level of Gli1 and Foxm1, (**E**) Gli1 expression level and CRC cells invasive capacity, (**F**) Foxm1 expression level and CRC cells invasive capacity analysed by Spearman's correlation test. (**G**) RNA and protein levels of Gli1 and Foxm1 in SCR, shGli1 cells and (**H**) SCRF, shFoxm1 cells. *p < 0.05, **p < 0.01, ***P < 0.001.

To explore the underlying relationship between Gli1 and Foxm1 in CRC, we compared Gli1 and Foxm1 levels in 126 tissues by qRT-PCR. We found that there were 25 low Foxm1 patients among 30 low Gli1 patients and 85 high Foxm1 patients among 96 high Gli1 patients. Obviously, Foxm1 was significantly positively correlated with Gli1 (*p <* 0.0001) (Figure 2A). To further approve the results, we analyzed both Gli1 and Foxm1 levels in LOVO, DLD1, Caco2, HT29, NCM460 cells by qRT-PCR and WB and found a positive correlation between Gli1 and Foxm1 expression (Figure 1C, Figure 2B, 2D). We also examined the invasive capacity of these cells and detected a positive correlation between Gli1, Foxm1 level and the invasive capacity (Figure 2C, 2E, 2F). Then, the Foxm1 level was down-regulated after Gli1 knockout in Lovo cells and this result from the opposite side showed the positive correlation between Gli1 and Foxm1 (Figure 2G).

According to the past studies, there were GLI-mediated genes constitute the positive or negative feedback loops in Hedgehog signaling cascade including PTCH1, PTCH2, HHIP1, BOC and etc [12]. To investigate whether feedback loops exist between Gli1 and Foxm1, we stably transfected Lovo cells with negative control vectors (SCRF) and Foxm1 knockdown lentivirus (shFoxm1). As showed in the Figure 2H, Gli1 expression had no statistical differences in these two interfered groups. So, we got a conclusion that there was no feedback loops between Gli1 and Foxm1.

### Gli1-Foxm1 axis decreased OS (Overall Survival) and PFS (Progression-free Survival) in CRC Patients

**Figure 3:**
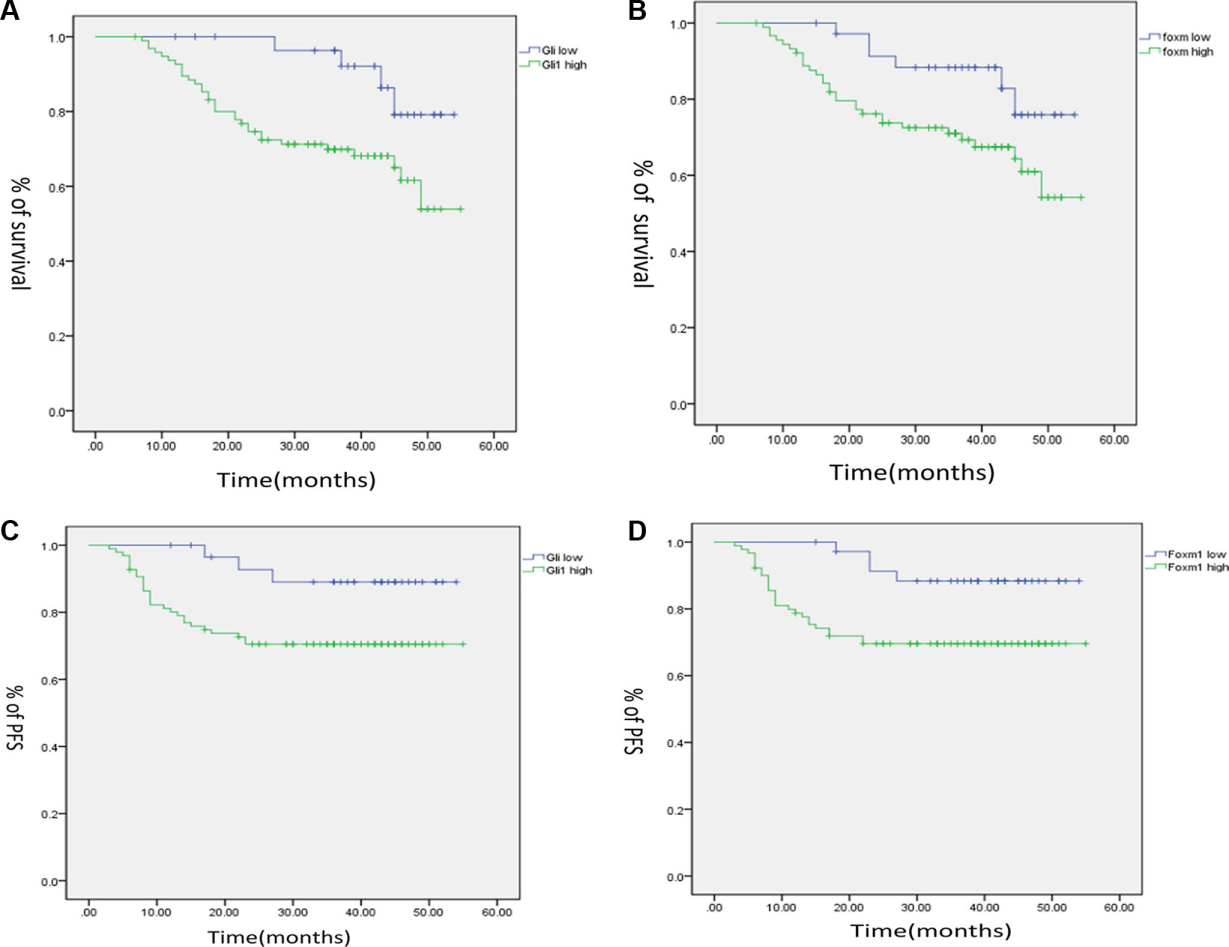
Kaplan–Meier analysis for the influence of Gli1 (**A**), Foxm1 (**B**) in overall. survival (OS), and Gli1 (**C**), Foxm1(**D**) in progression free survival (PFS) of CRC patients.

To explore the role of Gli1 and Foxm1 on the survival rate of CRC, we analysed the clinical outcomes of the patients. As shown by the Kaplan–Meier analysis, the OS of patients with high levels of Gli1 was lower than that of patients with low levels (*p* = 0.022; Figure 3A). In the similar way, high Foxm1 expression was also associated with poor OS in CRC patients (*p* = 0.04; Figure 3B).

There were also close connections between Gli1, Foxm1 and PFS. The Kaplan–Meier analysis showed that the PFS of patients with high Gli1 level was lower than that of patients with low Gli1 level (*p* = 0.033; Figure 3C). In addition, high Foxm1 level was also associated with poor PFS in CRC patients (*p* = 0.019, Figure 3D).

### Gli1 promotes CRC cells migration and invasion in a Foxm1-dependent manner *in vitro* and *in vivo*

**Figure 4:**
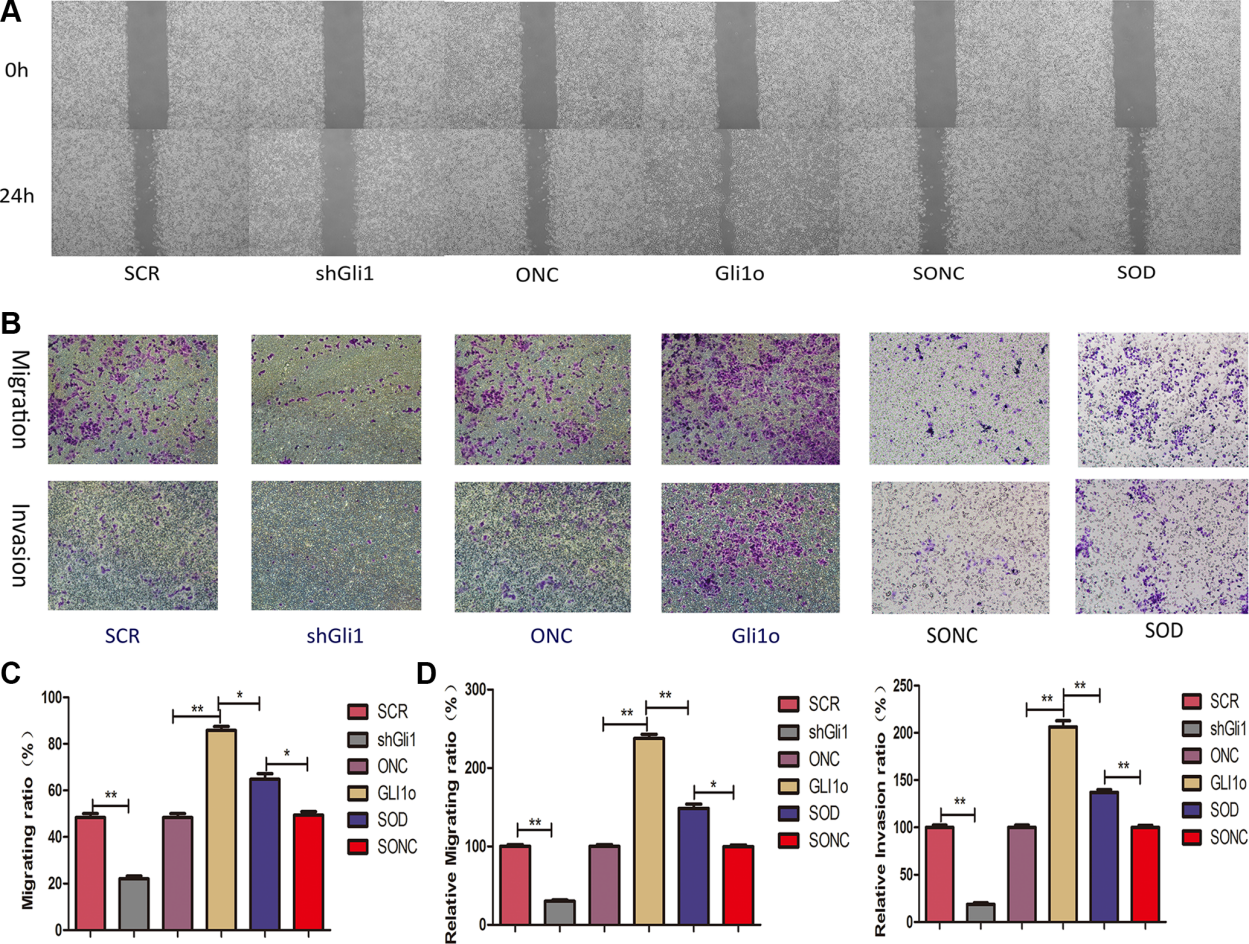
Gli1 promotes colorectal cancer cells migration and invasion in a Foxm1-dependent manner *in vitro*. (**A**) Wound-healing results and (**B**) Transwell assays results were performed *in vitro* by different types of Lovo cells. (**C**) Comparative analysis of migration ratio in different types of Lovo cells in Wound-healing. (**D**) Comparative analysis of relative migrating ratio and relative invation ration in different types of Lovo cells in Transwell assays.

In the wound healing assay (Figure 4A, 4C), we found that the closure of wound area of shGli1 was wider compared with SCR and ONC was wider than Gli1o. It was proved in the reverse that GLI1 can promote CRC cells migration. Similar results were obtained from the Transwell assay (Figure 4B, 4D) that transfection with shGli1 significantly suppressed the migration and invasion of Lovo cells from the upper side to the lower side and transfection with Gli1o had a reverse regulation. We also compared Glio with SOD and found that the migratory and invasive ability of SOD were weaker than Glio. So, knockdown of Foxm1 could mitigate the Gli1 overexpression effect on metastasis ability. We could get a conclusion that Foxm1 is an critical point in the progression of GLI1-Foxm1 axis regulating CRC cells migration and invasion *in vitro*.

**Figure 5:**
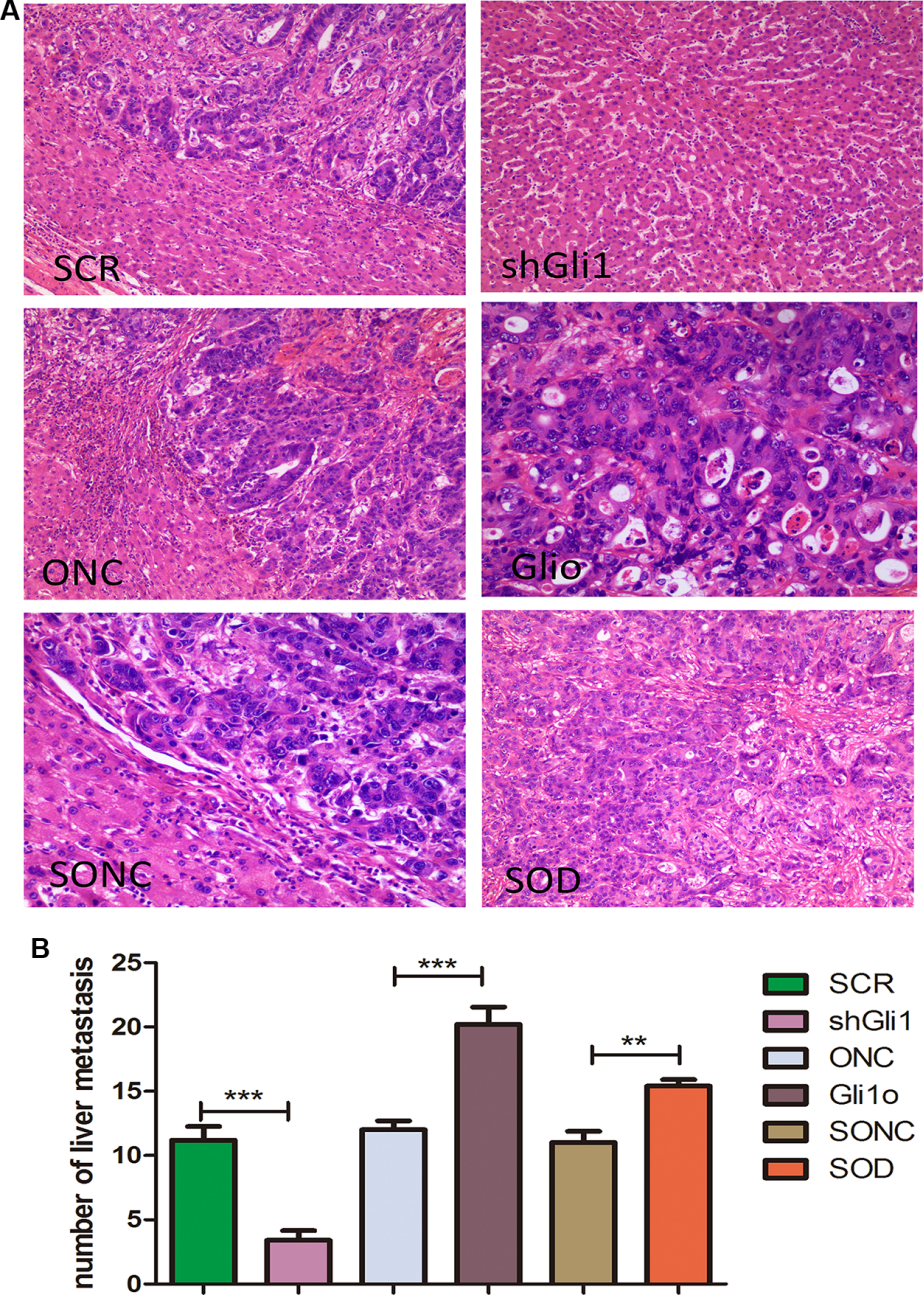
Gli1 promotes colorectal cancer cells migration and invasion in a Foxm1-dependent manner in vivo (**A**) HE staining results of the liver tissues from mice treated with different types of LOVO cells. (**B**) Comparative analysis of the number of the liver metastasis in mice treated with different types of Lovo cells. **p < 0.01, ***P < 0.001.

To confirm these findings *in vivo*, we carried out tail vein metastatic assays in nude mice using SCR, shGli1, ONC, Glio, SOD, SONC LOVO cells. At 7 weeks after injection, the results of HE staining (Figure 5) showed that high Gli1 expression has a close correlation with more liver metastases. And the SOD group results also confirmed the Foxm1, s role in the GLI1-Foxm1 axis. So, we concluded that Gli1 also promoted colorectal cancer cells migration and invasion in a Foxm1-dependent manner *in vivo*.

### Gli1-Foxm1 axis promotes EMT change in CRC cells

**Figure 6:**
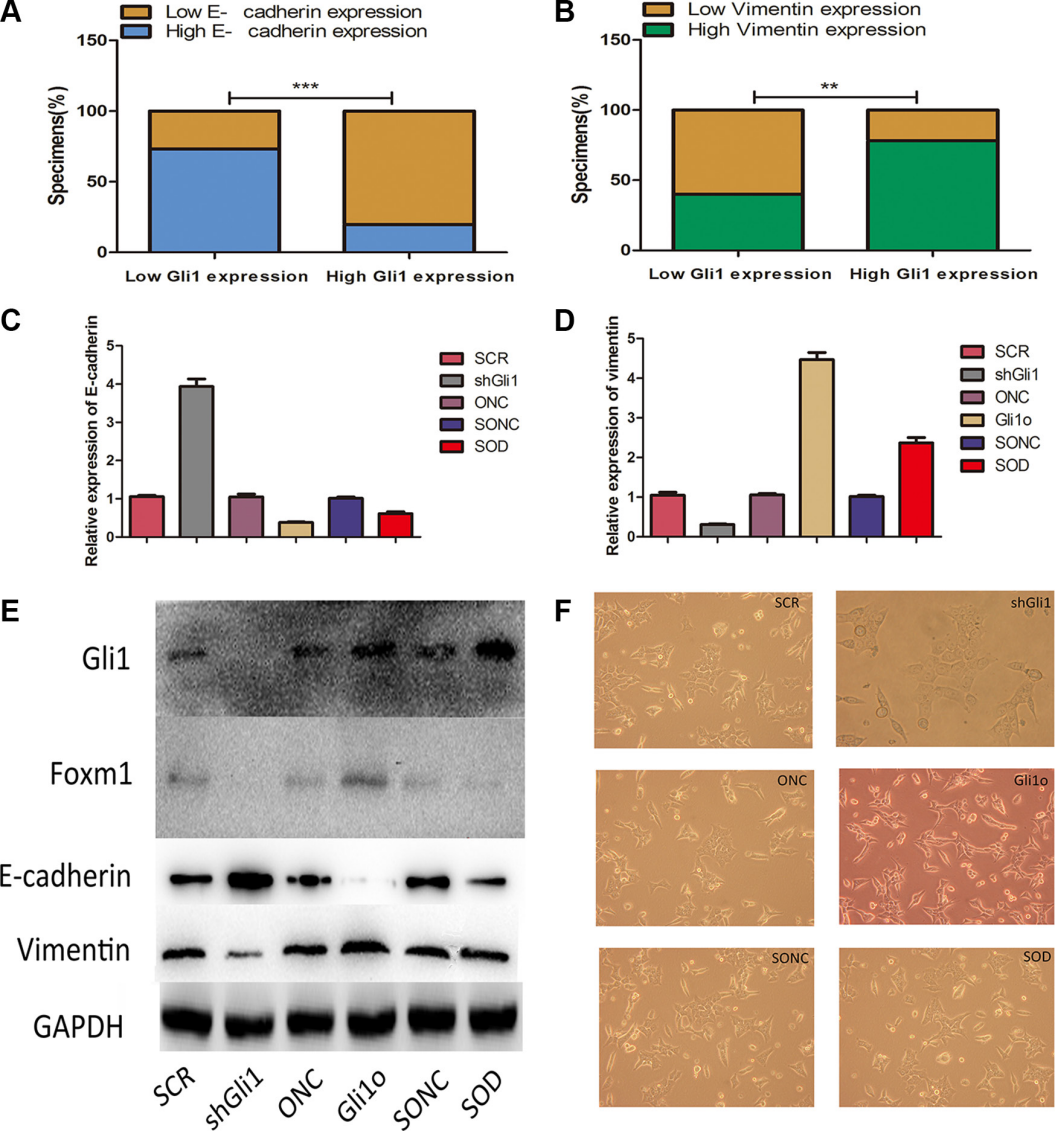
Gli1-Foxm1 axis promotes EMT change in colorectal cancer cells (**A**) Percentage of specimens exhibiting low or high Gli1 expression and association of Gli1 expression with expression levels of E-cadherin and vimentin (**B**) in CRC tumor specimens. **p < 0.01, ***p < 0.001. (**C**) E-cadherin and vimentin (**D**) expression levels were measured in different colorectal cancer cell lines by real-time PCR and western blot (**E**) together with Foxm1 protein expression levels in different type LOVO cells. The intensity of the bands was determined using densitometric analysis.**p < 0.01, *p < 0.05. (**F**) Morphology changes in in different type LOVO cells.

EMT (epithelial-to-mesenchymal transition), an essential cell biological program during embryonic development, contributes to cancer invasion and metastasis [13]. It regulates the phenotypic change from proliferating epithelial cancer cells to invading mesenchymal cancer cells, characterized by increased cell motility, resistance to genotoxic agents and so on [14]. There were some studies reported the crosstalk between hedgehog signaling and EMT in cancers like pancreatic cancer, bladder cancer, breast cancer, ect [15, 16]. However, there is no relevant research in this field in CRC. To understand whether there are any crosstalks between Gli1 regulating and EMT or not, we first examined the expression of EMT markers (E-cadherin, vimentin) in the tumor tissues by qRT-PCR and divided the patients into low level and high level. There were 22 high E-cadherin patients among 30 low Gli1 patients and 77 low E-cadherin patients among 96 high Gli1 patients (*χ*^2^ = 29.85, *P <* 0.05, Figure 6A); 18 low vimentin patients among 30 low Gli1 patients and 75 high vimentin patients among 96 high Gli1 patients (*χ*^2^ = 15.545, *P <* 0.05, Figure 6B). Obviously, the expression of E-cadherin was negatively correlated with Gli1 and vimentin was positively correlated with Gli1. To understand whether the Gli1-Foxm1 axis can regulate EMT progression in CRC cells, we evaluated the expression of Gli1, Foxm1, E-cadherin and vimentin at RNA and protein levels in Gli1o, ONC, SCR, shGli1 cells. The increased expression of E-cadherin and decreased expression of vimentin was detected in shGli1 cells and an inverse result in Gli1o cells (Figure 6C–6E). Consistent with the changes in genes expression, morphology changes also showed that Gli1 overexpression induce LOVO cells into scattered spindle-shaped invasive phenotype and Gli1 knockdown can induce adherent polygonal phenotype compared to the negative controls. This suggested that GLI1 can regulate the migration and invasion potential of the CRC cell lines by inducing EMT (Figure 6F).

To explore the role of Foxm1 in this progression, we evaluted the EMT markers in SOD and SONC cells by Western-blot. As showed in Figure 6C–6E, SOD had a similar result like SONC which was significantly different from Gli1o. The morphology changes also showed that Foxm1 knockdown reverted the EMT appearance in Gli1o Lovo cells (Figure 6F). Thus, Gli1-Foxm1 axis promotes EMT change in CRC cells.

### Integration of Gli1-Foxm1 axis and EGFR-PI3K/AKT signaling enhances colorectal cancer metastasis

Distorted epidermal growth factor receptor (EGFR) and its downstream signaling pathways are common events in the development of CRC [17]. The phosphatidylinositol 3 kinase (PI3K)/AKT pathway is one major intracellular pathway activated by EGFR [18]. The crosstalk between HH/Gli1 and EGFR signaling pathway was firsted studied in neocortical stem cells showing that Shh and EGF cooperate in the stimulation of cell proliferation [19]. And growing evidences showed that the internship of HH/Gli1 and EGFR-PI3K-AKT signaling played critical roles in increasing the occurrence and development of types of tumors [20, 21].

**Figure 7:**
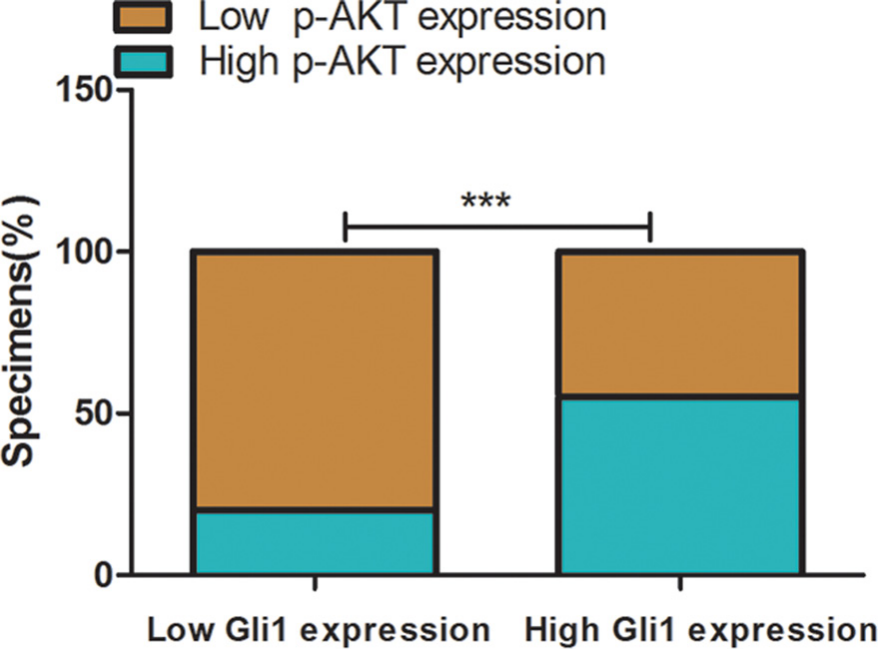
Percentage of specimens exhibiting low or high Gli1 expression and association of Gli1 expression with expression levels of p-AKT in CRC tumor specimens ***P < 0.001.

**Figure 8:**
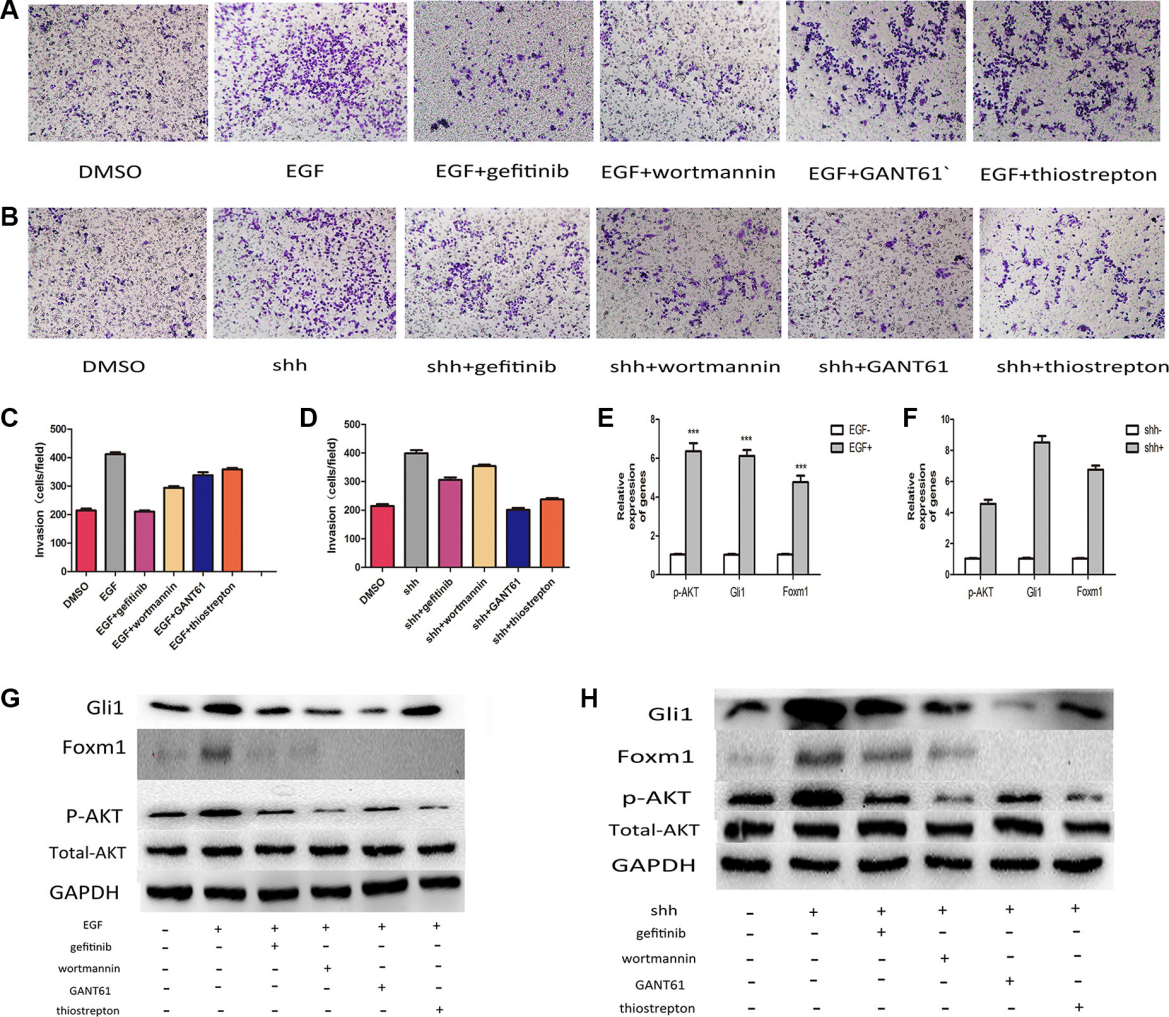
Integration of Gli1-Foxm1 axis and EGFR-PI3K/AKT signaling enhance colorectal metastasis (**A**) The invasive capacity of LOVO cells treated with EGF and EGF combined with different drugs was determined using invasion assays. (**B**) The invasive capacity of LOVO cells treated with N-shh and N-shh combined with different drugs was determined using invasion assays. (**C**, **D**) Comparative analysis of relative invation ration in Lovo cells treated with different drugs in Transwell assays. (**E**) p-AKT, Gli1, Foxm1 expresson levels analysed by qRT-PCR in LOVO cells treated with EGF or not. (**F**) p-AKT, Gli1, Foxm1 expresson levels analysed by qRT-PCR in LOVO cells treated with N-shh or not. (**G**, **H**) Comparative analysis of protein levels in Lovo cells treated with different drugs.

**Figure 9:**
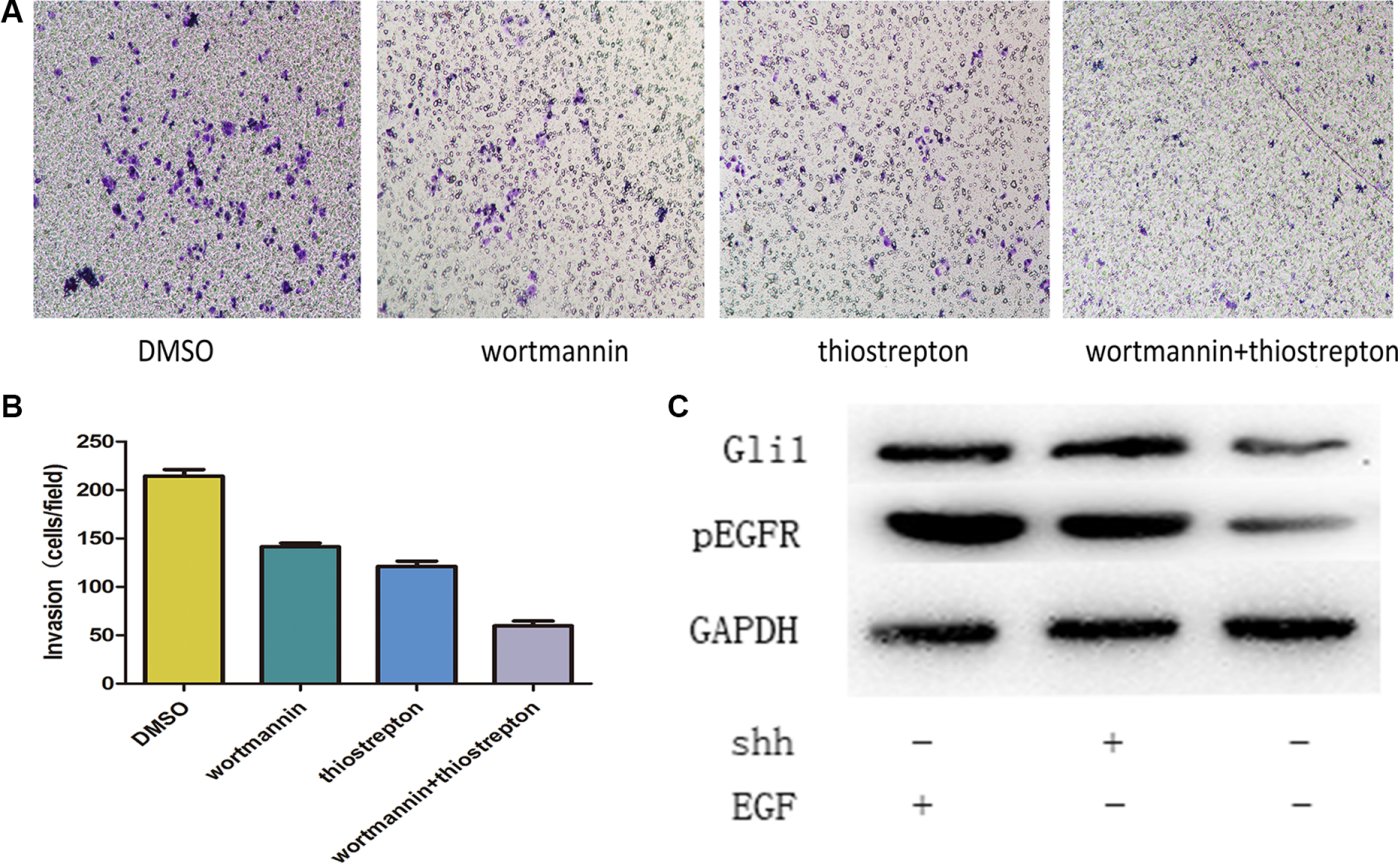
(**A**, **B**) Combined treatment with GANT61 and gefitinib reduced tumor cell invasion much more efficiently than the single drug. (**C**) Western-blot results showed that pEGFR overexpression was consistent with the Gli1 overexpression in N-shh group and a similar result in the EGF group.

In this study, we addressed whether integration of the Gli1-Foxm1 axis and EGFR-PI3K-AKT pathway is a critical step in colorectal cancer metastasis. We first examined p-AKT in the 126 tumor tissues and found that there were 24 low p-AKT patients among 30 low Gli1 patients and 53 high p-AKT patients among 96 high Gli1 patients (*χ*^2^ = 11.38, *P <* 0.05, Figure 7). Then, we used EGF (5 ng/ml for 1 h), N-Shh (5 μg/ml for 1 h) to induce the activation of AKT phosphorylation and Gli1-foxm1, separately. We found that both EGF and N-shh can promote the invasive and metastatic capacity of CRC cells significantly (Figure 8A–8D). In EGF group: the expression levels of p-AKT together with Gli1, Foxm1 all had an growth in different degrees and N-shh group showed a similar result (Figure 8E, 8F). To further verify whether Gli1/Foxm1 axis cooperate with EGFR-PI3K-AKT signaling or not, we blocked EGFR, PI3K, Gli1 and Foxm1 specifically by gefitinib, wortmannin, GANT61 and thiostrepton at IC50 concentration for 12 h, and 0.01% DMSO as a non-treated control. As invasion assays showed: In EGF group, gefitinib can reversed largely the EGF invasive promoting effect, while wortmannin, GANT61 and thiostrepton attenuated EGF effect in a faintish way. In the N-shh group, GANT61 played the maximum inhibitory role on N-shh invasive promoting effect and wortmannin, gefitinib, thiostrepton played inhibitory roles in different degrees (Figure 8A–8D). Additionally, thewestern-blot results also showed the corresponding gene expression changes (Figure 8G, 8H). We then treated Lovo cells by single gefitinib or GANT61 or a combination of them. As showed in invasion assays (Figure 9A, 9B), combined treatment reduced tumor cell invasion much more efficiently than single drug. These results illustrated that Gli1-Foxm1 axis and EGFR-PI3K-AKT pathway had an synergistic interaction in promoting the CRC cells metastastic capacity. But how did Gli1 participate into the EGFR-PI3K-AKT signaling process is still unknown. As reported, EGF binding to EGFR leads to the phosphorylation of specific cytoplasmic tyrosine residues of EGFR, including positions 992, 1068, 1086, 1148 or 1173 [22]. The pEGFR (phosphorylated epidermal growth factor receptor) result in activation of a series of pathways then and the increased Akt phosphorylation is consistent with the increase in Y1068 phosphorylation mostly [23]. Thus, we speculated that Gli1 participates into AKT signaling by promoting pEGFR-Y1068 expression. We detected pEGFR-Y1068 protein in CRC cells treated with N-shh or EGF. Western-blot results showed that pEGFR overexpression was consistent with the Gli1 overexpression in N-shh group anda similar result in the EGF group (Figure 9C). So, Gli1-Foxm1 axis activated Akt signaling by promoting pEGFR-Y1068 expression and Akt signaling stimulated Gli1-Foxm1 axis by increasing Gli1 expression.

## DISCUSSION

Hh signaling drives cell proliferation, promotes cell survival, and directs cell differentiation in the developing embryo [24]. Recent studies have shown that aberrant signaling of this pathway is involved in a variety of human cancers, such as basal cell carcinomas, medulloblastomas and small-cell lung cancer [25, 26]. Gli1 has been proved to be an indispensible gene of Hh signaling and play important roles in types of tumor progressions [11, 27]. But, it is controversial in CRC metastasis. Thus, in our study, we focused on the biologic mechanism of Gli1-Foxm1 axis in the metastasis of CRC by investigating the expression of GLI1 and Foxm1 in CRC tissues and cell lines.

Our results showed that Gli1 was overexpressed in CRC tissues and its overexpression was also found to be closely correlated with the metastasis factors. Foxm1, which is a downstream target of Gli1, was found to have a positive correlation with the Gli1 level both in CRC tissues and cells. And these two genes levels were positively related with the CRC cells invasive capacity. The first innovative finding of our study was that we used gene interfering and cell functional examinations to get a negative result of feedback loop between Gli1 and Foxm1. Not like the genes like PTCH1, PTCH2, HHIP1and BOC had feedback loops with Gli1 [28–30].

The second innovative point was that we retrospectively analysed the clinical outcomes of 126 patients and found that high Gli1 and Foxm1 could cause poor OS and PFS. In this study, we only observed the liver metastasis but not other organs in mice because 40–50% CRC patients died from liver metastasis and we emphasisd more on the poor prognosis of liver metastasis in the clinical works [31]. In the coming studies, based on the fingings about liver metastasis, we may continue to explore Gli1-Foxm1 in other organs metastasis.

Epithelial-mesenchymal transition (EMT) is an important step in embryogenesis and cancer progression. The transition requires the activity of complex functional networks that downregulate properties of the epithelial phenotype and upregulate characteristics of the mesenchymal phenotype [16]. In our study, the EMT markers expression, invasion capacity together with the cell morphology changes in response to Gli1 changes indicated that Gli1 had a significant impact on the process of EMT by mediating E-cadherin and vimentin in CRC cells. In the further research, we will continue to examine Gli1-Foxm1 and EMT in more deep level *in vivo*. On the other hand, our study also digged out the mechanisms between Gli1-Foxm1 axis and EGFR-PI3K/AKT signaling in promoting the CRC cells invasion ability by treating them with different reagents and drugs from different angles. These results herald the crosstalk between Gli1-Foxm1 axis and EGFR-PI3K/AKT in colorectal cancer is in existence and also provide a new idea for targeted therapy in clinical work. These two exploration results are the third innovative point of our study. In the coming studies, we plan to do some intuitionistic molecules researches on these genes to investigate the cooperation more deeply.

In summary, an increased expression of Gli1 and Foxm1 was observed in CRC tissues and cell lines. More importantly, the expression level had a positive correlation with metastatic potential of primary tumor. Additionally, Gli1 promoted CRC cells migration and invasion in a Foxm1-dependent manner *in vitro* and *vivo*. And the crosstalk between Gli1-Foxm1 axis and EMT, EGFR signaling provided new visual fields on the tumorigenesis, progression and therapy of CRC metastasis.

## MATERIALS AND METHODS

### Patients and tissue specimens

We collected primary tumor tissues and the corresponding adjacent normal tissues of 126 nonselected patients diagnosed as CRC in our hospital between March 2010 and January 2011. The normal tissue was extracted > 5 cm from the edge of the tumor. Samples were frozen in liquid nitrogen within 5 minutes after resection. None of the participants had a history of cancer or had received radiotherapy, chemotherapy, radiofrequency ablation or other treatments before surgery. The clinical stages and pathologic features were defined according to the criteria of the American Joint Commission on Cancer. All the patients were treated according to the NCCN guildline. The median duration of follow-up after a curative resection was 34.4 ± 12.95 months.

### Cell lines and cell culture conditions

Human colorectal carcinoma LOVO, CACO2, DLD1 and HT29 cell lines and normal colon epithelial NCM460 cell line were bought from ATCC (American Type Culture Collection, Manassas, VA, USA) and preserved in our institute. Cells were cultured in DMEM medium containing 10% fetal bovine serum (Gibco, Grand Island, NY, USA) and 50 U/ml penicillin and streptomycin at 37°C in an atmosphere of 5% CO2.

### Quantitative real-time PCR (qRT-PCR)

Total RNA was isolated from tissues or cell cultures using TRIzol reagent (Invitrogen, USA) according to the manufacturer's protocols and cDNA was synthesized using Primescript RT kit (Takara, Japan). Quantitative real-time PCR was performed on an Applied Biosystem 7500 Real-time PCR system using SYBR-Green Master (Roche). The specific oligonucleotide primer sequencesare listed in Table 3. GAPDH was used as an internal control and the qRT-PCR result was quantified by 2–ΔΔCT method.

**Table 3:** Oligonucleotide primer sequences used in the qRT-PCR

Gene	Primer sequence
FOXM1	F: 5′-GGAGCAGCGACAGGTTAAGG-3′
FOXM1	R: 5′-GTCGTGCAGGGAAAGGTTGT-3′
GLI1	F: 5′-TCTGCCCCCATTGCCCACTTG-3′
GLI1	R:5′-TACATAGCCCCCAGCCCATACCTC-3′
E-cadherin	F: 5′-GACCGAGAGAGTTTCCCTACG-3′
E-cadherin	R: 5′-TCAGGCACCTGACCCTTGTA-3′
Vimentin	F: 5′-ATGACCGCTTCGCCAACTAC-3′
Vimentin	R: 5′-CGGGCTTTGTCGTTGGTTAG-3′
p-AKT	F: 5′-GACTACCTGCACTCGGAGAAG-3′
p-AKT	R: 5′-TGTGATCTTAATGTGCCCGTC-3′
GAPDH	F: 5′-AGAAGGCTGGGGCTCATTTG-3′
GAPDH	R: 5′-AGGGGCCATCCACAGTCTTC-3′

### Lentivirus packaging and stable transfection cell line generation

Lentiviral constructs was designed by the Genepharma (Shanghai). The cancer cells were stably transfected with Gli1 overexpression (Gli1o) lentivirus and LV5-EF1a-GFP-Puro negative control vectors (ONC). For Gli1 knockdown, the cells were stably transfected with LV3-pGLV-h1-GFP-puro negative control vectors (SCR) and Gli1 knockdown lentivirus (shGli1). Briefly, Foxm1 knockdown was shFoxm1 and negative control was SCRF. Gli1 overexpression combined with Foxm1 knockdown was SOD and the negative control was SONC. Target cells (1 × 10^5^) were transfected at a lentivirus/medium ratio of 1:50 in the presence of 5 μg/ml polybrenein. Each experiment was conducted three times, and data were averaged.

### Wound-healing assay

Colorectal carcinoma cells were seeded in 6-well plates with a density of 2 × 105 cell/well and grown to confluency. Then pipette tips of 200 μl (Corning, USA) were used to scrape over the adherent cells to make a wound. 0 and 24 h after wounding, migrating cells at the wound front were photographed using an inverted microscope. We used imagepro-plus 6.0 to calculate the migrating ratio (migrating ratio = [Average width of linear wound at 0 h-Average width of linear wound at 24 h]/Average width of linear wound at 0 h). Each experiment was performed at least three times in triplicate.

### Cell invasion and migration assays

Cell invasion and migration assays were performed by using transwell chamber (8 mm, 24-well format; Corning) which was coated with or without diluted Matrigel (BD Biosciences). Cells (1.5 × 104) were suspended in 100 μl of serum-free medium and placed in the upper compartment of each chamber and 600-μL culture medium with 10% serum was added into the lower chamber as a chemoattractant. The chamber was cultured in 37°C, 5% CO2 incubator for 24 h. Cotton swab was used to remove reduced cells in the top chamber, and then, the chambers were fixed with methanol. Cells outside the inserts were stained with 1% crystal violet for 20 min. The number of invaded cells on the membrane was then counted under a light microscope and the results are calculated as means ± SD. This assay was performed three times.

### Western blot analysis

Western blot analysis was performed as described previously [32]. In this experiment, primary antibodies includedGli1, Foxm1 (1:1000, Santa, Cruz); E-cadherin, vimentin, total AKT (C67E7), phospho-Akt (Ser473), GAPDH (1:1000; Cell Signaling Technology); pEGFR-Y1068 (1:1000; Abcam). GAPDH was used as an internal loading control.

### Chemicals

EGF and N-shh was purchased from Thermo Scientific. GANT61 (120904), Wortmannin (ab120148), Thiostrepton (ab143458) were purchased from Abcam. Gefitinib (ZD1839, S1025e) were purchased from Selleck Chemicals. All chemicals were dissolved in dimethylsulfoxide (DMSO; Sigma-Aldrich) and were used at the indicated concentrations.

### Immunohistochemistry (IHC)

Immunohistochemistry was performed as described preciously [33]. Primary antibody used Gli1 (1:200; Santa Cruz). IRSs (IRSs, immunoreactive scores) was the product of the percentage of positive cells (0, < 5%; 1, 5%–25%; 2, 25%–50%; 3, 50%–75%; 4, > 75%) multiplied by the staining intensity (0, negative; 1, weak; 2, moderate; and 3, strong). The IRS was then classified as negative (0–1), weakly positive (2–3), moderately positive (4–7), or strongly positive (8–12).

### Tail vein metastatic assay

Thirty nude mice (15 females and 15males) aged 5 to 6 weeks were purchased from Animal Center of Nanjing medical university and raised in the Animal Laboratory again. We diveded the mice into 6 groups:SCR, shGli1, Gli1o, ONC, SOD, SONC. Then, 2 × 10^6^ cells suspended in 200 μl of PBS were injected into the mouse tail vein. The liver tissues were removed after 7 weeks and stained with haematoxylin and eosin (HE). All experimental procedures were approved by the Experimental Animal Welfare and Ethics Committee of Nanjing Medical University.

### Statistical analysis

Statistical analysis was performed with the SPSS software 20.0. All the data were expressed as the means ± standard deviation (SD). Fisher's exact test was used to assess the relationship between Gli1 expression and each clinicopathological factor and the correlation between genes and Gli1 expression. Cumulative survival analysis was done using the Kaplan–Meier method and analyzed by the log-rank test. Multivariate analysis was done using the Cox proportional hazard model. *χ*^2^ test was used to assess clinical information of the patients in two groups. The *t* test was performed to assess the differences in total cell count. A *p value* of less than 0.05 was considered significant in all analyses.
